# Long-term stress in dogs is related to the human–dog relationship and personality traits

**DOI:** 10.1038/s41598-021-88201-y

**Published:** 2021-04-21

**Authors:** Amanda Höglin, Enya Van Poucke, Rebecca Katajamaa, Per Jensen, Elvar Theodorsson, Lina S. V. Roth

**Affiliations:** 1 IFM Biology, Linköping University, 581 83 Linköping, Sweden; 2 Division of Clinical Chemistry, Department of Biomedical and Clinical Sciences, Faculty of Medicine and Health Sciences, Linköping University, 581 85 Linköping, Sweden

**Keywords:** Animal physiology, Animal behaviour

## Abstract

Previously, we found that dogs belonging to the herding breed group, selected for human cooperation, synchronise their long-term stress levels with their owners. The aim of the current study was to investigate features that could influence long-term stress levels in ancient dog breeds, genetically closer to wolves, and dogs specifically selected to work independently of their owner. Twenty-four ancient breed dogs and 18 solitary hunting dogs were recruited and hair samples were obtained from both dogs and owners from which hair cortisol concentration (HCC) was analysed. Additionally, the owners completed lifestyle surveys, the Monash Dog Owner Relationship Scale (MDORS) on human–dog relationship, and both dog and owner personality questionnaires (Dog Personality questionnaire and Big Five Inventory survey). The results from the MDORS indicate that the subscale *Perceived cost* correlated to the dog HCC of tested breed groups: solitary hunting breeds (χ^2^ = 4.95, P = 0.026, β = 0.055), ancient breeds (χ^2^ = 2.74, P = 0.098, β = 0.027), and herding dogs included from a previous study (χ^2^ = 6.82, P = 0.009, β = − 0.061). The HCC of the solitary hunting dogs was also related to the owner personality traits *Agreeableness* (χ^2^ = 12.30, P < 0.001, β = − 0.060) and *Openness* (χ^2^ = 9.56, P = 0.002, β = 0.048) suggesting a more substantial influence of the owner on the solitary hunting dog’s HCC compared to the ancient breeds. No effect of owner HCC on dog HCC was found in either ancient or in solitary hunting breeds. Hence, the long-term stress synchronisation is likely to be a trait in breeds selected for human cooperation. In conclusion, dog HCC is often related to the owners’ personality, but is primarily influenced by the owner-dog relationship.

## Introduction

In a previous study we found that dogs mirror the long-term stress levels of their owners^1^. The HCC of the dog was shown to synchronise with the HCC of the owner and was in addition more associated to the owner’s personality compared to its own. Emotional contagion has earlier been shown through both short-term physiological, endocrine, and behavioural responses between dogs and humans^2–4^ and could be a result of the domestication of the dog and sharing everyday life. It might, however, also be affected by the recent breed selection and the working task of the breed. Both border collies and Shetland sheepdogs revealed a strong long-term stress synchronisation with their owners^1^. However, both belong to the herding breed group, specifically selected to cooperate with humans, and this might have contributed to the stress synchronisation with the owner. Recent selection of breeds, and even breed lines within breeds^5^, has been shown to affect the behaviour of dogs^6^, and this might also have an effect on the interspecies emotional contagion. In contrast to herding dogs, the ancient breeds are thought to be closer genetically to wolves^7^ and have not been selected specifically for human cooperation. Also, there are those breeds that are selected for hunting independently, visually separated from the owner, and that have been shown to differ in both their attention as well as behaviour towards humans^8,9^. During hunting seasons in Sweden, there is a tradition of releasing hunting dogs into the forest to let them work on their own. The dog will search the terrain, find targeted animals and drive them forward or, alternatively, make them stationary and gain the hunter’s attention through barking. Hence, these dogs are not specifically bred to cooperate with humans but instead bred for their natural hunting skills. In a recent study^10^ the degree of selection of coopertiveness with humans was shown to affect stress-related behaviours during owner separation. Therefore, we aimed in this study to assess long-term stress levels in hair samples from dogs and their owners, from both solitary hunting dogs as well as ancient dog breeds, and compare the results to our previous study on herding dogs^1^.

Hair cortisol concentrations (HCC) have repeatedly been shown to be a valuable long-term measurement in various mammals and humans^11,12^ since the cortisol is incorporated as the hair grows. However, HCC can also be affected by e.g. physical activity^13^ which might differ between the e.g. solitary hunting breeds and other breed groups. Therefore, we continuously also monitored the dogs activity levels during one week using remote cloud-based activity collar. Since our previous study^1^ found effects of owner personality traits on the dogs’ HCC, personality surveys were also completed.

Interestingly, human–dog interactions and aspects of the human–dog relationship, including owners’ perceptions of the costs of dog ownership, have previously been shown to be associated with alterations in plasma oxytocin concentrations^14^. In the current study, we are interested in whether there is also an effect of relationship features on long-term stress levels, as measured from cortisol incorporated in hair. Therefore, the owners of ancient dog breeds and solitary hunting dog breeds included in this study, in addition to the owners of herding breeds of the previous study^1^, were asked to complete the validated Monash Dog Owner Relationship Scale (MDORS)^15^.

Hence, our aim was to reveal features that might affect dog HCC in dogs that were not selected for human cooperation. In other words, whether the owner’s long-term stress, her/his perceived relationship with the animal, and personality traits affected the dog HCC. Our hypotheses were that a stronger owner-dog relationship might have stronger association with the HCC of the dog, but also that the degree of selection for human cooperation might affect the dog-owner long-term stress synchronisation. Therefore, we recruited dogs of ancient breeds and dog actively used to hunt independently of humans, and asked the owners to provide hair samples and complete surveys about their personalities and human–dog relationship.

## Material and methods

### Subjects

The participants were recruited through social media, and personal contact with e.g. breeders and hunting associations. Dogs of ancient breeds (N = 24, 15 females and 9 males), according to genetic data^7^, and solitary hunting breeds (N = 18, 14 females and 4 males) were recruited with their owners (owners of ancient dog breeds: 18 females and 6 males; owners of hunting dog breeds: 10 females and 8 males). Informed consent was obtained from all dog owners for participating in the study along with their dogs.

### Hair cortisol analysis

This study is part of a larger project that also includes behavioural experiments, and therefore all subjects were invited to Linköping University, in Southeast Sweden. During this visit (September–October 2019) we obtained hair samples from both owners and their dogs. The hair sampling and analysis of hair cortisol concentration (HCC) were performed as described previously in Sundman et al.^1^. In short, hair was cut from the dog’s neck and from the posterior vertex area of the owner’s head and the two most proximal centimeters of hair were cut into smaller pieces. Approximately 7 mg was weighed (for later calculations of concentration cortisol per milligram hair), homogenized and extracted with methanol overnight. Then, 0.8 mL of the methanol supernatant was pipetted off and lyophilized and the remaining pellets was dissolved in RIA buffer in order to perform the Radioimmunoassay (RIA) analasis.

### MDORS

The owners completed the MDORS^15^ about their relationship to their dog. The MDORS is a 28-item questionnaire which generates scores for three different sub-scales for each human–dog dyad: *MDORS 1-Dog-Owner Interaction*, *MDORS 2-Perceived Emotional Closeness*, and *MDORS 3-Perceived Costs*. In addition to the owners of the ancient dog breeds and solitary hunting dogs, we asked the dog owners from a previously published study to complete the same MDORS survey. Those dogs were border collies and Shetland sheepdogs, both belonging to the herding breed group^1^.

### Personality surveys

Personality of the owners was assessed using a Big Five Inventory survey (BFI)^16^. The survey consisted of 44 statements that were rated on a 5-point Likert scale, and assessed the following personality traits: *Extraversion*, *Agreeableness*, *Conscientiousness*, *Neuroticism,* and *Openness*. The personality of the dogs was determined through the validated Dog Personality Questionnaire (DPQ)^17^, which was completed by the owners. It consisted of 75 questions rated on a 5-point Likert scale and resulted in five different factors: *Activity/Excitability*, *Responsiveness to training*, *Aggression towards people*, *Aggression towards animals*, and *Fearfulness*.

### Physical activity

Thirty-six dogs (20 dogs of ancient breeds and 16 hunting dogs) were equipped with a PetPace smart collar for seven days during September–November. Three days (including one weekend day) were randomly chosen and further analysed. The collar, which is a wireless cloud-based collar, collected activity data continuously from accelerometer outputs which resulted in activity scores every second minute. The activity scores were categorised into rest, low, medium, and high according to the PetPace algorithm and transformed into a percentage for each dog.

### Lifestyle features

The owners completed a survey about their everyday life. The survey included Likert scale questions about how much they play with their dog, reward/correct their dog, but also if they have children in the household, if the dog spends time in an outdoor kennel, whether the owner works full-time or not, if they have other animals in the household, and if they have participated in competition/s during the last two years. We also asked how often they spent time together in nature, but due to low variation (since they all did this almost every day), this question was not used. However, it mirrors the active life of included dogs.

### Data analysis

Before analysing the HCC results, extreme outliers determined by SPSS (more than three interquartile ranges) were removed to account for analysis errors or other possible molecular interactions with antibodies in the RIA. The distribution of neither ancient nor hunting breeds HCC met the criteria for normal distribution (Shapiro Wilk, P < 0.001) and a Kruskal–Wallis test was used when comparing HCC between breed groups since we also included HCC of the herding dog breeds from our previous study^1^.

The Generalized linear models (GLMs) testing the effect of MDORS on dog HCC consisted of MDORS scores (covariates) for the three subscales (*Dog–Owner Interaction*, *Perceived Emotional Closeness*, and *Perceived Costs*) using gamma distribution with log link function. The breed groups were tested separately and we also performed GLMs for the herding breed group^1^ using normal identity model type with log link due to data distribution. In addition, MDORS scores for all three breed groups (ancient, hunting, and herding) were compared using Kruskal–Wallis tests since the subscale scores were non-normally distributed (Shapiro Wilk, P < 0.1). All pairwise comparisons for the subscales were adjusted by the Bonferroni correction for multiple tests. For the comparison between companion and competing herding dogs, Mann–Whitney U tests were used. In addition, GLMs were used to test the associaton between owner and dog personality traits (co-variates) on the MDORS scores using gamma distribution with log function.

In the GLMs testing the effect of human HCC and personality, the ancient and hunting breed groups were, again, tested separately and gamma distributions were chosen with the log link function. When testing the effect of human HCC (covariate), sex of the dog and owner were treated as fixed factors and interactions between the covariate and the fixed factors were also included in the model. When testing dog and human personality traits, the traits were treated as covariates, and sex of the dog and owner as fixed factors. Since the personality data consisted of data that was both normally- and non-normally distributed (according to Shapiro–Wilk tests), personality comparisons between the ancient breed group, the solitary hunting breed group, and the herding breed group (from our previous study Sundman et al.^1^) were performed using non-parametric Kruskal–Wallis tests. All pairwise comparisons were adjusted by the Bonferroni correction for multiple tests.

Due to the distribution of the HCC data, Spearman correlations were used for dog HCCs and activity measurements (PetPace data) where medium and high activity scores were summed for each breed group. According to Shapiro–Wilk (ancient P = 0.19, hunting P = 0.19, herding P = 0.063) the activity data was not different from normal distribution and the comparison between groups was performed using One-Way Anova.

Likert scores (1–7) from the lifestyle questionnaire were used to group participating dogs into two groups for each question. If the question concerned positive aspects (e.g. how often do you play with your dog, where 1 = not at all and 7 = several times a day), a score of 5–7 was considered as often. However, if the question considered negative aspects (e.g. how often do you correct your dog) a score of 4–7 was considered as often in order to account for the subjectivity of the responses. Similarly, from a multiple choice question, owners that work full-time were separated from others (part-time, on parental leave, retired etc.). For the GLMs, we used gamma distribution with the log link function, and the breed groups were tested separately. In the GLMs that tested the effect of lifestyle, the following features were considered as fixed factors: Other dogs/cats in the household (or none), Children up to 13 years old in the household (or none), Competing (or not), Spending time in outdoor kennel (or not), Playing often with the dog, Rewarding the dog often, Correcting the dog often, and Working full-time (or not).

For all GLMs, Akaike’s information criterion was used to determine the best fit of the model, and all statistical analyses were performed using IBM SPSS software (version 26). All data and subject information can be found in Supplementary Table S1 and the model information can be found in Supplementary Table S2.

### Ethical note

Ethical approvals were obtained from the regional ethical committee for animal experiments (Permit number: 6065/2019) with the inclusion of human subjects (Permit number: 2019-01796) in Linköping, Sweden. All methods were performed in accordance with the relevant guidelines and regulations and all dog owners were informed and gave their written consent that they voluntarily participated in the study.

## Results

### Owner and dog personality

**Figure 1:**
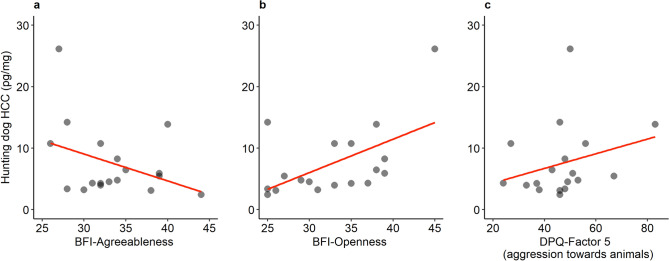
Correlations with fitted lines between dog HCC (pg/mg) of solitary hunting breeds’ and the human personality trait BFI-Agreeableness (**a**), BFI-Openness (**b**) and the dog personality trait DPQ Factor5-Aggression towards animals (**c**). This image was created using R version 4.0.4. https://www.R-project.org and package ggplot2 https://ggplot2.tidyverse.org.

The HCC of three females of ancient dog breeds, were removed due to extreme outlier values. GLMs were used to analyse the associations between human and dog personality traits and dog HCC. For ancient dog breeds (N = 21) *DPQ Factor 2-Aggression towards people* (χ^2^ = 3.47, P = 0.063, β = − 0.063) tended to affect dog HCC even if it did not reach statistical significance. For the solitary hunting dog breeds (Fig. 1, N = 18), *BFI-Agreeableness* (χ^2^ = 12.30, P < 0.001, β = − 0.060), *BFI-Openness* (χ^2^ = 9.56, P = 0.002, β = 0.048), and *DPQ Factor 5-Aggression towards animals* (χ^2^ = 9.19, P = 0.002, β = − 0.051) were all significantly associated to the dog’s HCC.

**Figure 2:**
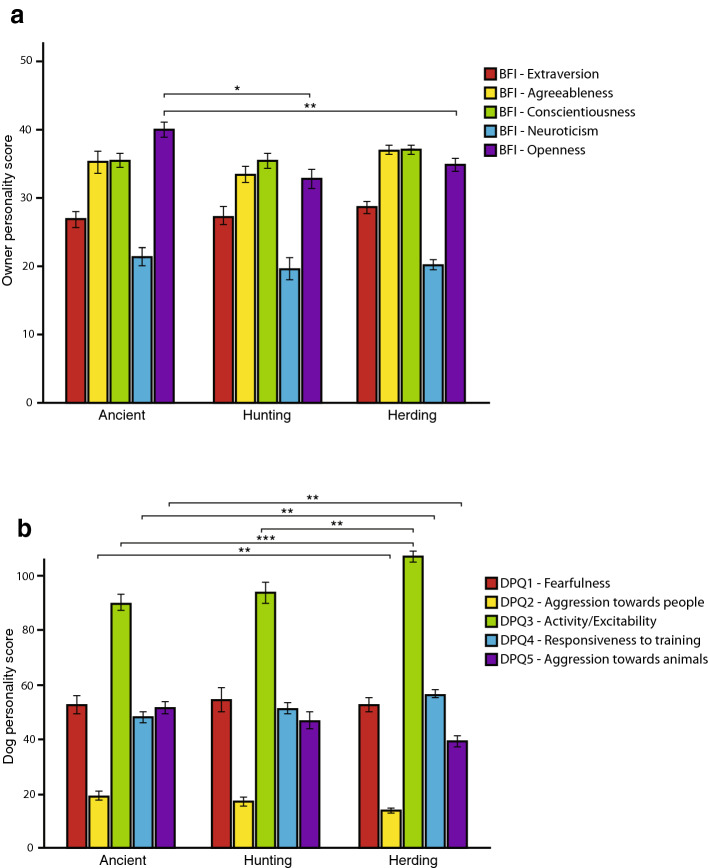
Mean personality scores ± SE for owners (**a**) and dogs (**b**) belonging to ancient breeds, solitary hunting breeds and herding breeds. Significant differences are indicated by *p < 0.05, **p < 0.01, ***p < 0.001. This image was created using Adobe Illustrator 2021, version 25.2.1, https://www.adobe.com.

When comparing the owner personality traits between breed groups, we also included the herding dogs from a previous study (N = 57) and compared all three groups (Fig. 2a). The only trait that was significantly different between breed groups was *BFI-Openness* (χ^2^ = 12.14, P = 0.002), where owners of ancient breeds had higher scores than those of both hunting (P = 0.007) and herding breeds (P = 0.025). The trait *BFI-Extraversion* was not significant (χ^2^= 3.24, P = 0.198), but there was a tendency for *BFI-Agreeableness* to differ between the breed groups (χ^2^ = 5.88, P = 0.053). The traits *BFI-Conscientiousness* (χ^2^ = 4.19, P = 0.123), and *BFI-Neuroticism* (χ^2^ = 1.14, P = 0.565) were similar between groups.

We also included the herding dogs (N = 58) when comparing the dog personality traits (Fig. 2b), which revealed significant differences between breed groups for all traits except for *DPQ Factor1-Fearfulness* (χ^2^ = 0.081, P = 0.961). *DPQ Factor2-Aggression towards people* was significantly different (χ^2^ = 13.60, P = 0.001), where pairwise comparisons showed that ancient breeds had significantly higher scores than herding breeds (P = 0.003). *DPQ Factor3-Activity/Excitability* was significantly different (χ^2^ = 19.25, P < 0.001), and here both ancient (P < 0.001) and hunting breeds (P = 0.023) revealed lower scores than herding breeds. *DPQ Factor4-Responsiveness to training* differed between groups (χ^2^ = 16.07, P < 0.001), where ancient breeds had significantly lower scores than herding breeds (P = 0.001). The last *DPQ Factor5-Aggression towards animals* also differed between breed groups (χ^2^ = 17.42, P < 0.001) and here the ancient breeds had significantly higher scores than herding breeds (P < 0.001; Fig. 2b).

### Human–dog relationship and its associations with dog HCC

In addition to participating dogs of ancient (N = 24) and solitary hunting breeds (N = 18), owners belonging to the herding breed group (N = 36) from our previous study^1^ were asked to complete the MDORS about their relationship with their dog.

**Figure 3:**
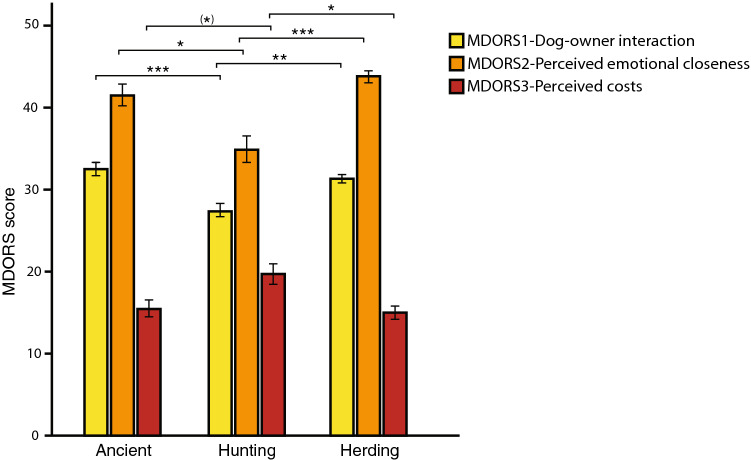
Mean MDORS scores ± SE for the ancient breeds, solitary hunting breeds and herding breeds. Significant differences are indicated by (*)p < 0.06, *p < 0.05, **p < 0.01, ***p < 0.001. This image was created using Adobe Illustrator 2021, version 25.2.1, https://www.adobe.com.

Interestingly, we found differences between the three breed groups for all three MDORS Subscales (N = 78, Fig. 3); *MDORS 1-Dog–Owner Interaction* (χ^2^ = 19.81, P < 0.001), where pairwise comparisons revealed significant differences between ancient and hunting breeds (P < 0.001), and also between herding breeds and hunting breeds (P = 0.003); *MDORS 2-Perceived Emotional Closeness* (χ^2^ = 19.32, P < 0.001), where again ancient and hunting breeds differed (P = 0.013), and also herding breeds and hunting breeds (P < 0.001); and lastly *MDORS 3-Perceived Costs* (χ^2^ = 10.35, P = 0.006), where ancient and hunting breeds tended to differ (P = 0.053) even if it did not reach statistical significance, and, again there was a significant difference between herding breeds and hunting breeds (P = 0.012). Hence, ancient and herding dyads were similar in their MDORS scores, while dyads with solitary hunting breeds showed an opposite pattern (Fig. 3).

Herding dogs consisted of both companion dogs (N = 18) and competing dogs (N = 18), but there was no difference in MDORS scores between these lifestyle groups (*MDORS 1-Dog–Owner Interaction* U = 136.5, P = 0.424; *MDORS 2-Perceived Emotional Closeness* U = 125.5, P = 0.252; *MDORS 3-Perceived Costs* U = 151.0, P = 0.743).

**Figure 4:**
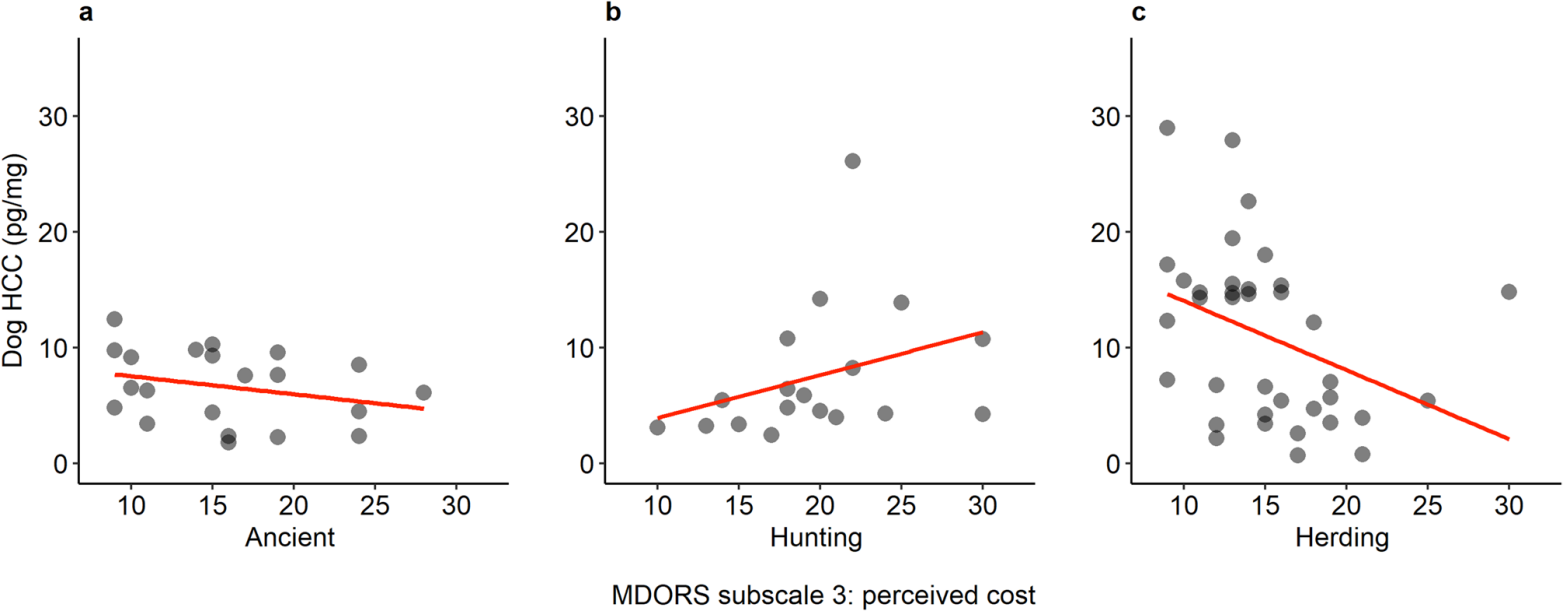
Correlations with included fitted lines between dog HCC (pg/mg) and MDORS 3*-*Perceived cost for ancient breeds (**a**), solitary hunting breeds (**b**) and herding breeds (**c**). This image was created using R version 4.0.4. https://www.R-project.org and package ggplot2 https://ggplot2.tidyverse.org.

GLMs were used to analyse the associations between MDORS subscales, dog sex, and the HCC of ancient breeds, solitary hunting breeds, and herding dogs separately (Fig. 4). In ancient breeds (N = 21 after removal of three HCC outliers), *MDORS 2-Perceived Emotional Closeness* (χ^2^ = 2.93, P = 0.087, β = − 0.060) and *MDORS 3-Perceived Cost* (χ^2^ = 2.74, P = 0.098, β = − 0.027) tended to affect the dogs’ HCC even if it did not reach statistical significance. In the solitary hunting breeds (N = 18), *MDORS 3-Perceived Cost* (χ^2^ = 4.95, P = 0.026, β = 0.055) was significantly associated with the dogs’ HCC, where there was an increase in HCC with an increase in MDORS score. Sex of the hunting dogs also had a significant effect (χ^2^ = 5.60, P = 0.018) where female dogs (N = 14, mean = 8.55 ± 1.70 pg/mg) had higher HCC than males (N = 4, mean = 4.00 ± 0.28 pg/mg), but no interactions between sex and MDORS subscales were found. In herding dogs (N = 36), there was again a significant association between the dogs’ HCC and *MDORS 3-Perceived Cost* (χ^2^ = 6.82, P = 0.009, β = -0.061), but here an increase in HCC was accompanied with a decrease in the MDORS score (Fig. 4).

### Associations between MDORS and personality

GLMs were used to study the associations between MDORS subscale scores and both owner and dog personality traits. *MDORS 1-Dog–Owner Interaction* tended to be associated to *BFI-Neurotisism* (χ^2^ = 2.90, P = 0.088, β = 0.007). *MDORS 2-Perceived Emotional Closeness* were significantly associated with *DPQ Factor 2—Activity/Excitability* (χ^2^ = 9.68, P = 0.002, β = 0.012) and showed a tendency to be related to *BFI-Conscientioiusness* (χ^2^ = 3.41, P = 0.065, β = 0.005). Lastly, *MDORS 3-Perceived Costs* revealed associations with *BFI-Conscientioiusness* (χ^2^ = 6.13, P = 0.013, β = − 0.017), *DPQ Factor 1—Fearfullness* (χ^2^ = 3.90, P = 0.048, β = 0.015), and *DPQ Factor 4—Responsiveness to training* (χ^2^ = 3.89, P = 0.049, β = -0.004).

### Owner HCC, sex, and breed group associations

The HCC of three female dogs of ancient breeds and two female owners were removed due to extreme outlier values. GLMs were used to analyse the associations between owner HCC, and sex of the dog/owner on dog HCC for each breed group separately. There were no significant effects for the ancient breed group (N = 20). For the solitary hunting breeds (N = 18) there was an association between dog sex and dog HCC (χ^2^ = 6.62, P = 0.010) as mentioned before, where the females (N = 14, mean = 8.55 ± 1.70 pg/mg) had higher HCC than males (N = 4, mean = 4.00 ± 0.28 pg/mg).

When comparing possible breed group differences in dog HCC we also included the herding dogs from our previous study^1^. However, there were no significant differences in HCC between ancient breeds (N = 21, mean = 6.61 ± 0.68 pg/mg), solitary hunting breeds (N = 18, mean = 7.54 ± 1.39 pg/mg), and herding dogs (N = 58, mean = 10.91 ± 0.84 pg/mg; χ^2^ = 3.17, P = 0.205).

### Lifestyle features

We used GLMs to analyse possible associations between dog HCC and general lifestyle aspects, but none of the included features (working status, the use of outdoor kennels, children in the household, other cats/dogs in the household, playing/rewarding/correcting often, or competing with the dog) had an effect on either the ancient dog breeds (N = 21), or the solitary hunting dog breeds (N = 18).

### Physical activity

There were no significant correlations between each dog’s summed medium and high physical activity data, obtained by the PetPace collar, and dog HCC for either the ancient dog breeds (N = 18, Rs = − 0.084, P = 0.74), or the solitary hunting dog breeds (N = 16, Rs = -0.032, P = 0.91).

We compared possible breed group differences in percentage of medium and high physical activity in ancient breeds (N = 20 mean = 13.64 ± 0.73), solitary hunting breeds (N = 16 mean = 14.82 ± 0.91), and herding breeds (N = 46, mean = 16.01 ± 0.80), but no significant differences were found (N = 82, F = 1.85, P = 0.164).

## Discussion

In our previous study we found dogs of the herding breed group to synchronise with their owners in long-term stress^1^. The aim of this study was to investigate dogs that are not selected or used specifically for human cooperation in order to reveal possible origins of this, so far unique, interspecies stress contagion, but also to further investigate features affecting dog HCC. Interestingly, we found that, independently of breed group tested in this study, the owner-dog relationship was associated with the dog HCC. Owner personality traits influenced the solitary hunting breeds more than ancient breeds, but there were no indications of long-term stress synchronisation between owners and their dogs. Instead, the long-term stress contagion found between herding dogs and their owners^1^ might be due to the breed selection for close human cooperation, while dog HCC is related to the owners personality to various degrees depending on breed group.

### Personality

For the ancient breeds, regarded to be genetically closer to the wolf^7^, we did not detect any association between owner personality traits and their dogs’ HCC. The HCC of participating hunting breeds, selected to hunt independently from humans, showed a negative association with the trait *Agreeableness* and a positive association with *Openness,* suggesting a larger influence of the owner on the hunting dogs’ HCC compared to the ancient dog breeds. In our previous study on border collies and Shetland sheepdogs, both belonging to the herding breed group, several owner personality traits were associated with their dogs’ HCC but there was little association with dog personality traits^1^. Dog personalities related to aggression where the only traits that had an effect on dog HCC in this study, were the ancient breeds and hunting breeds showed similar scores for most traits. Interestingly, social and non-social fear traits have recently been shown to correlate negatively with HCC in border collies^18^, suggesting that stress-related personality traits such as fear and aggression influence dog HCC.

### Human–dog relationship

For both ancient and hunting breeds in this study, and herding breeds in our previous study^1^, the owner-dog relationship was associated with the dogs’ HCC, though this was less apparent in the ancient breeds. Hence, HCC in dogs of different breed groups might be affected by the owner and the human–dog relationship to different degrees. In addition, complicating it further, personality traits might influence the dog-owner relationship. In our study it was mostly dog personality traits that was associated with MDORS and few owner personality traits. Previous studies have suggested that owner personality has stronger associations with the dog-owner relationship compared to dog personality^19^. Hence, future studies will need to disentangle this possible triadic association. Hoummady and colleages^20^ recently revealed associations between human and working dog personalities, their relationship and the dyad’s search performance. However, only binary data such as “toys at home” and the use of punishment were used to measure the human–dog relationship, and future studies would benefit from a more detailed approach, and possibly also from measurements that take the dogs perspective of the human–dog relationship into account.

Our comparison between the breed groups in this study and our previous study^1^, regarding the owner perceived human–dog relationship (MDORS), revealed similar results for ancient and herding dogs. In contrast, owners of solitary hunting breeds experienced less positive interactions with their dogs, perceived a weaker emotional bond, and considered their dogs as being more costly compared to owners of ancient or herding breeds. These results might mirror the purpose of the dogs participating in the study. While the ancient group of dogs in this study were generally kept as companion dogs, the hunting group of dogs were to a large extent kept for the purpose of hunting. However, the MDORS subscales did not differ between companion and competing herding dogs, suggesting a more complex association.

In line with our results, a higher emotional closeness in the human–dog relationship was previously reported by owners of dogs showing more social fear/aggression^19^. In this study, the ancient dog breeds that were reported to have high scores on social aggression, also had higher scores for emotional closeness compared to the solitary hunting dogs. Still, the MDORS subscale with the strongest association with dogs’ HCC in this study was the subscale for *Perceived cost*. A low score for the *Perceived cost* subscale has previously been associated with high oxytocin concentrations in blood^12^, which could decrease cortisol concentrations^21^. This was also in accordance with the HCC results for the solitary hunting dogs in this study. However, for the herding breed group, which had the lowest score for *Perceived cost*, the direction of association was reversed and it revealed instead a decrease in HCC with increased *Perceived cost* scores. Hence, a multifaceted approach including associations between breed selection, personality, and human–dog relationship is needed in order to untangle the long-term endocrine responses of the dog.

### HCC synchronisation

As mentioned before, our results show little support for long-term stress synchronisation between owners and their ancient breed or solitary hunting dog breeds. Hence, the synchronisation found in herding dogs^1^ is probably not a result of the domestication process, but could have emerged due to the selection for human cooperation. We did find an effect of dog sex in the dog HCC of the solitary hunting dog breeds, where females had higher HCC than males. However, the result should be interpreted cautiously since there were few males in the solitary hunting breed group. Still, in our previous study we found female dogs to have higher HCC and to synchronise more with their owner than the males^1^, but other studies on German Shepherd dogs, Labrador retriever dogs and border collies did not find sex differences in the dog HCC^22^.

### Limitations

The owners were recruited on a voluntary basis through social media and personal contacts and are therefore biased towards enthusiastic owners interested in science. This bias might increase the similarities between the included breed groups and owner personality traits, and there is a possibility that we therefore e.g. underestimate the differences.

All included hunting dogs in this study were actively used for hunting due to their specific skills to hunt independently of humans, and these dogs are rarely (allowed to be) bought as companion dogs. Hence, lifestyle and environmental features could differ between our breed groups in addition to their genetic background, which might have affected their long-term stress level and human–dog relationship. This was found for the herding dogs where competing dyads revealed a stronger long-term stress synchronisation than dyads with companion dogs^1^. However, the MDORS results did not differ between companion and competing herding dogs. And, similar to our previous study^1^, we found no effect of lifestyle features such as working status, spending time in outdoor kennel, or children in the household. Lifestyle and relationship data were only collected once in the current study, but future studies would benefit from a more longitudinal approach, following dyads over years during both good and bad times. Hunt and colleageus^23^ investigated dyads during three years and found e.g. human depression to be associated with stress-related problems in the dog, but the illness of the dog was also a stressor for the human.

Also, for future studies on dog HCC it could be interesting to include e.g. an additional hunting group with breeds selected to hunt in collaboration with humans e.g. spaniels and retrievers, then it would be possible to also include a group from the same breeds used solely as companion dogs (since the solitary hunting breeds are primarily used actively for hunting and not solely for companionship). These breeds are also more common than solitary hunting dogs which could facilitate a larger number of included dogs.

### Conclusions

The human–dog relationship may influence the HCC of the dog and especially the subscale for Perceived cost. Comparing the dog HCC between breed groups suggests that the ancient breed group is the least affected by the owner and their relationship together, the solitary hunting dogs show clear associations with both owner personality traits and their relationship, but only the herding dogs show interspecies long-term stress synchronisation. Hence, these results suggest that the long-term stress synchronisation is influenced by the recent selection for human cooperation, but that the human–dog relationship and personality traits are important features affecting dog HCC.

## Supplementary Information

Supplementary Information 1.

Supplementary Information 2.
